# Investigation of Phosphatidylcholine by MALDI Imaging Mass Spectrometry in Normal and IVF Early-Stage Embryos

**DOI:** 10.3390/ijms25137423

**Published:** 2024-07-06

**Authors:** Stefánia Gitta, Éva Szabó, Alexandra Sulc, Péter Czétány, Gábor Máté, András Balló, Tímea Csabai, Árpád Szántó, László Márk

**Affiliations:** 1 Department of Analytical Biochemistry, Institute of Biochemistry and Medical Chemistry, Medical School, University of Pécs, 7624 Pécs, Hungary; stefania.gitta@aok.pte.hu (S.G.); sulc.alexandra@pte.hu (A.S.); 2 National Laboratory on Human Reproduction, University of Pécs, 7621 Pécs, Hungary; czetany.peter@pte.hu (P.C.); mate.gabor@pri.hu (G.M.); ballo.andras@pte.hu (A.B.); szanto.arpad@pte.hu (Á.S.); 3 Urology Clinic, Clinical Center, University of Pécs, 7621 Pécs, Hungary; 4 Pannon Reproduction Institute, 8300 Tapolca, Hungary; 5 Institute of Biology, Medical School, University of Pécs, 7624 Pécs, Hungary; timi.csabai@gmail.com; 6 Imaging Centre for Life and Material Sciences, University of Pécs, 7624 Pécs, Hungary; 7 HUN-REN-PTE, Human Reproduction Research Group, 7624 Pécs, Hungary

**Keywords:** embryo transfer, endometrium, imaging mass spectrometry, in vitro fertilization, MALDI, mouse, phospholipids, pregnancy

## Abstract

The receptive phase of the uterus is marked by structural and functional maturation of the endometrium. During this limited time span, the blastocyst competency is superimposed on the receptive endometrium. It is a well-known fact that lipid signalling in early-stage pregnancy has a crucial role in successful embryogenesis. In our study, CD-1 mouse uteri after normal and in vitro fertilization (IVF) were investigated at 6.5, 8.5, and 10.5 days of pregnancy. Matrix-assisted laser desorption/ionization time-of-flight imaging mass spectrometry and liquid chromatography coupled tandem mass spectrometry were used for identification of phosphatidylcholine (PC) lipid structures. In the embryonal tissues, PC 32:0 and PC 34:0 were increased, while in the antemesometrial (AM) decidua the two 20:4-containing PCs, PC 36:4 and PC 38:4 were increased. In transferred uterus samples, higher expressions of PC 34:0, PC 34:1, PC 34:2, PC 36:1, and PC 36:2 in mesometrial decidua were seen, whereas the two 20:4-containing PCs, PC 36:4 and PC 38:4 showed increased expression in the AM and lateral decidua. This paper shows a significant spatio-temporal change in lipid metabolism during IVF procedures for the first time.

## 1. Introduction

Infertility is a worldwide problem affecting over 186 million people, or about 8–12% of couples in their reproductive age [1]. International data reported different effectiveness over the world in 2011, with the highest pregnancy rate per aspiration in North America (40.2%) and the lowest in Asia (17.1%) [2]. Although assisted reproduction technologies (ARTs) are improving, pregnancy per aspiration and live birth per aspiration are still low. In the background, several factors stand out, like impaired endometrial receptivity, asynchrony between the endometrial stage, and embryo development as a result of disturbed hormonal responses during ovarian stimulation.

Lipids have several diverse functions in the human body. They store energy in the form of triacylglycerols, and they build up cell membranes as structural lipids forming a phospholipid (PL) bilayer that separates the cytoplasm from the extracellular space. Lipids also serve as intracellular messengers, like eicosanoids derived from arachidonic acid (AA, C20:4n-6). About 93% of the total AA in the uterus is stored in PLs, 80% of which is stored in phosphatidylcholine (PC), phosphatidylethanolamine (PE), and phosphatidylserine (PS) [3]. From the PLs of the membrane, AA is released by the enzyme phospholipase A2. AA is used by cyclooxygenase (COX) enzymes to produce prostaglandins, which belong to the eicosanoid molecules. Several types of prostaglandins produced in this way (PGI2, PGE2, PGF2α) play important roles in ovulation, fertilization, implantation, and decidualization, which represent morphological and functional changes in endometrial cells, complemented by maternal artery remodelling [4]. Primarily, prostacyclin (PGI2) is essential for implantation and decidualization, which exerts its effect by activating one type of peroxisome proliferator-activated receptor (PPARβ/δ). Activation of the receptor initiates cell proliferation and angiogenesis, which are essential processes in the early embryogenesis phase [5].

Another important molecule is lysophosphatidic acid (LPA), which, as a signalling molecule, plays an important role beyond implantation in the equidistant embedding of blastocysts in the uterus. This process is called spacing. Exactly how this process takes place is currently only an assumption. According to this, LPA induces intermittent contraction of the uterine myometrium [6]. The expression of one of the oestrogen- and progesterone-regulated G-protein-coupled receptors (LPAR3) is particularly elevated during the peri-implantation period, which coincides with increased prostaglandin synthesis. This may be due to the fact that LPAR3 indirectly enhances the function of the COX-2 enzyme. This presupposes a very close interaction between the two mentioned signalling pathways [4,7].

As the different lipid classes have different functions and many of them have only a short-term effect causing quick change in their spatio-temporal distribution, an increasing number of studies are focusing on this topic. There are a growing number of studies investigating the role of lipids in oocytes [8,9,10], early stages of embryo development [10,11,12,13], placenta in late pregnancy [14], as well as endometrial tissues during the window of implantation [15], and endometrial fluids [16].

Several previous studies have investigated the role of lipids in in vitro fertilization (IVF) in the peri-implantation period. A study of women undergoing IVF found a correlation between peri-implantational endometrial lipids and an early rise in progesterone, suggesting a possible link between endometrial receptivity and endometrial lipid concentrations [15]. In addition, follicular fluid also showed increased glycerophospholipid metabolism, which, through incomplete oxidation and lipotoxicity, may result in lower quality oocytes during IVF [17]. Several lipids in the follicular fluid have also been suggested as potential biomarkers in female-related infertility [18], and the follicular fluid lipidome [19] or even the serum lipidome [20] might help predict later pregnancy outcomes in IVF. In the endometrial fluid just before embryo transfer, some PC and PE lipids were significantly decreased in non-implantation cycles, suggesting another important role of lipids in successful implantation [16]. Not only do lipids appear to be involved in proper implantation and in predicting the success of IVF, but this relationship appears to be bidirectional: IVF may cause a different lipid profile in the placenta than in a normal pregnancy [14], and even in foetal liver samples of the offspring [21].

In an animal study, early implantation was studied in a mouse model with matrix-assisted laser desorption/ionization time-of-flight mass spectrometry (MALDI TOF MS) imaging and the dynamic change of lipids was described [4]. In our previous systematic review, we showed that in the peri-implantation period several PLs, mainly PCs, show spatial and temporal changes, in both maternal and embryonal tissues [22]. Previous research has suggested that there may be differences between the lipidomics of IVF and normal pregnancy, but to our knowledge no study has investigated the spatial and temporal changes in lipids in the peri-implantation period in both the uterus and the implanting embryo.

Therefore, the aim of our study was to investigate the effects of IVF on the PC lipids of both maternal and embryonal tissues with mass spectrometry imaging (MSI) during early pregnancy and compare it to normal pregnancy in a mouse model.

## 2. Results

**Figure 1:**
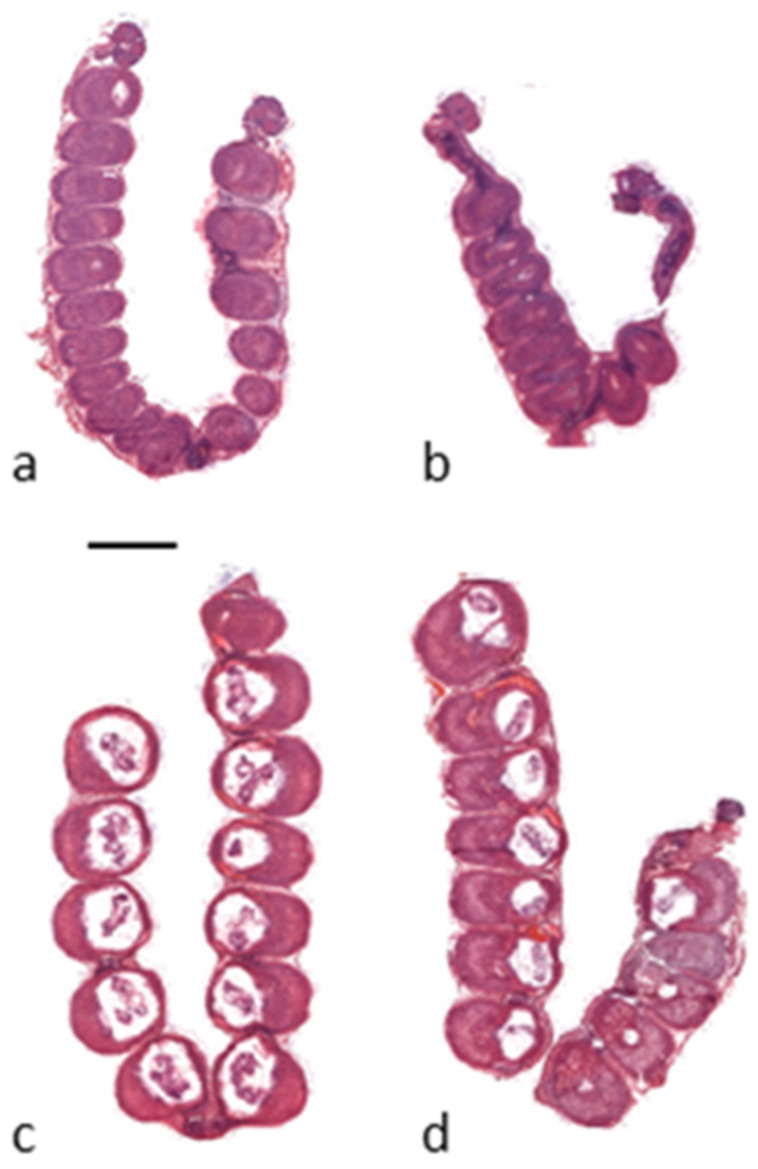
Conventional (haematoxylin and eosin stained) histological longitudinal sections of normal pregnant mouse uteri and embryos at 8.5 (**a**) and 10.5 embryonic days (**c**) and after embryo transfer in IVF pregnant mouse uteri and embryos at 8.5 (**b**) and 10.5 embryonic days (**d**). Scale bar: 5000 μm.

Fewer embryos were seen in the IVF pregnant uterus than in the normal pregnant uterus. There was also a spatial difference: in IVF pregnancies, the positioning of the embryos was also disturbed, with less space between two implanted embryos compared to a normal pregnancy. Figure 1 shows these differences between normal and IVF pregnancies at embryonic day 8.5 and 10.5 on conventional histological sections. In the case of IVF embryos, the implantation sites in the uterus are not developed symmetrically (Figure 1b,d), and the rate of spontaneous abortions are significantly higher than in normal pregnancies.

### 2.1. Differences between Normal and IVF Pregnancies in the Distribution of PCs

**Figure 2:**
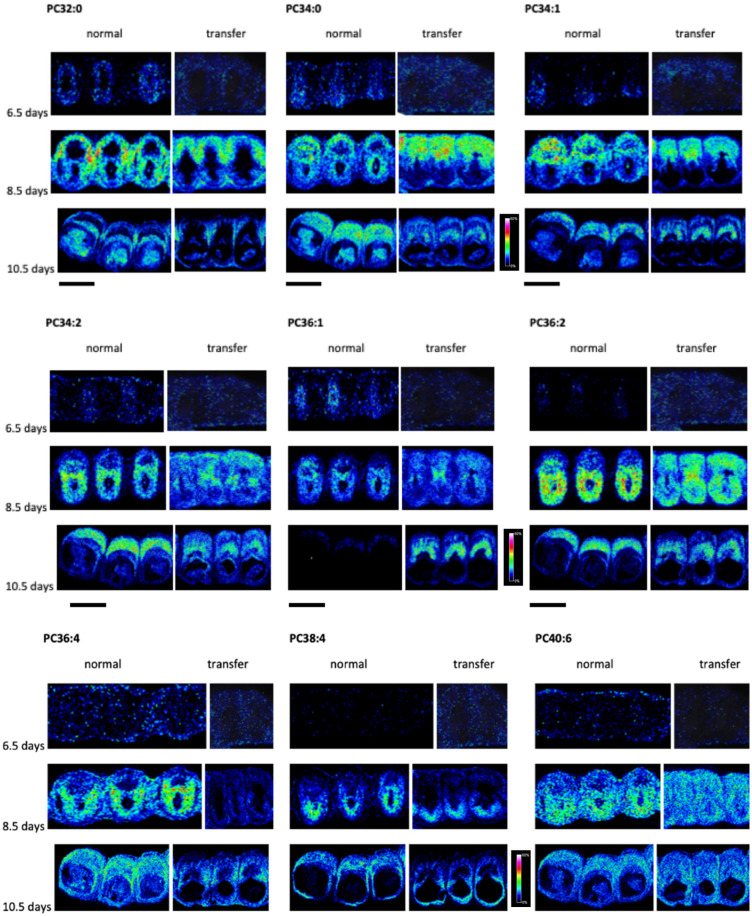
Spatial and temporal change of some PC lipids by MALDI imaging mass spectrometry in normal pregnancy or after embryo transfer in the embryo-containing uterus at embryonic day 6.5, 8.5, and 10.5. All scale bars: 2000 μm (Colour code: blue, low concentration; red, high concentration).

PLs are of vital importance during embryogenesis. In this study, we focused on the major PLs in cell membranes, the PC species, and investigated them by MALDI imaging mass spectrometry. In the spatio-temporal distribution, significant changes were detected between normal and IVF pregnancies at all time points examined. The summarized results are shown in Figure 2. For the identification of the resulting PC species protonated, sodium and potassium adduct ions as precursors and a specific fragment of phosphocholine ion (*m*/*z* 184) were detected by LC-MS/MS in all cases.

Briefly, at embryonic day 6.5 of the normal pregnancy, we found an increase for PC 32:0 in uterine stromal cells at implantation sites except for the primary decidual zone (PDZ). PC 34:0, PC 34:1, and PC 34:2 (Supplementary Figures S1–S4) showed higher expression in uterine stromal cells at implantation sites, while PC 36:1 and PC 36:2 were higher in the PDZ (Supplementary Figures S5 and S6). In contrast, in embryo-transferred animals the PCs showed an increase in glandular epithel at inter-implantation sites.

On day 8.5 of the normal pregnancy, PC 32:0, PC 34:0, and PC 34:1 showed higher levels in the mesometrial pole (M-pole), while PC 34:2, PC 36:2, PC 36:4, PC 38:4, and PC 40:6 showed higher levels in the antimesometrial pole (AM-pole). In contrast, in IVF uterus samples PC 32:0, PC 34:0, PC 34:1, and PC 34:2 were increased in the M-pole; PC 36:4 and PC 38:4 in the AM-pole; while PC 36:2 and PC 40:6 showed higher expression in both the M-pole and AM-pole (Supplementary Figures S1–S9).

At day 10.5 of the normal pregnancy, PC 32:0 showed higher intensity in the placenta; PC 34:1, PC 34:2, PC 36:2, and PC 40:6 in the mesometrial decidua; while PC 34:0 and PC 36:4 were higher in both placenta and mesometrial decidua. In the embryonic tissues, PC 32:0 and PC 34:0 were increased, whereas in the AM decidua, the two AA containing PCs, PC 36:4 and PC 38:4, were increased. In transferred uterus samples, a higher expression of PC 34:0, PC 34:1, PC 34:2, PC 36:1, and PC 36:2 in mesometrial decidua was seen, whereas PC 36:4 and PC 38:4 showed increased expression in the AM and lateral decidua. At the embryo absorption sites, we saw a substantial increase in PC 36:2 in the M-pole and PC 38:4 in the AM-pole (Figure 2, Supplementary Figures S1–S9).

### 2.2. Temporal Change of PCs in Normal and IVF Pregnancies

In both normal and IVF pregnancies, we observed alterations in the lipid composition of individual anatomical structures, as well as temporal variation in the PC lipids investigated (Figure 2). In normal pregnancies, for example, PC34:2, PC36:2, and PC36:4 exhibited higher intensities, primarily at the AM pole on embryonic day 8.5. However, two days later, on embryonic day 10.5, the intensity of these lipids was highest in the mesometrial decidua. On embryonic day 6.5, the intensity of PC36:4, PC38:4, and PC40:6 was low. However, on embryonic day 8.5, they exhibited higher intensities at the AM pole, primarily in the secondary decidual zone. In contrast, two days later (10.5 days) the intensity of these compounds decreased, with the maximum intensity observed at the mesometrial decidua. A similar temporal change was observed in the investigated PCs in IVF pregnancies. For instance, PC36:1 showed low intensities at embryonic days 6.5 and 8.5, whereas the highest expression was observed in the mesometrial decidua at embryonic day 10.5. PC34:0 and PC34:1 were observed to have low intensities at day 6.5, with an increase in the M-pole at embryonic day 8.5, and two days later, the highest intensity was seen at the mesometrial decidua (Figure 2).

## 3. Discussion

**Figure 3:**
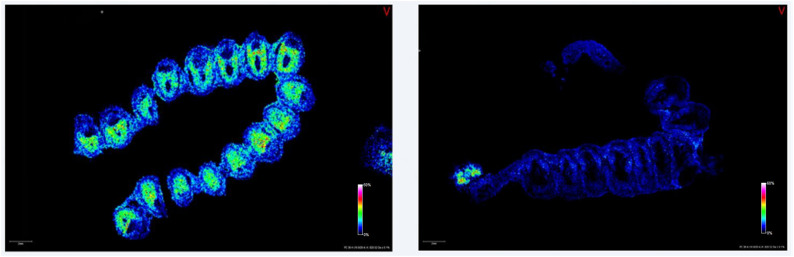
Representative local distribution of PC 36:4 by MALDI imaging mass spectrometry at 8.5-day-old normal (**left**) and IVF (**right**) pregnancies. PC 36:4 was located at the antimesometrial zone in the normal pregnancy and almost absent in IVF embryos, but highly expressed in the ovarium. Scale bars: 2000 μm (Colour code: blue, low concentration; red, high concentration).

To the best of our knowledge this is the first study investigating the effect of IVF on the lipids of foetal-maternal tissues during early pregnancy. In a previous study, a spatio-temporal change of lipids in the normal early pregnancy was detected [4]. With MALDI TOF imaging, an intensive dynamic change was noticed in a day-to-day approach, raising the importance of different lipid classes in the window for implantation. Several studies have suggested that in IVF cases the lipids in the preimplantation embryo may be different from normal controls, raising the question that this altered embryonal lipid composition caused by the IVF might affect the implantation too [23,24,25,26,27,28]. On the basis of these previous studies, our aim was to investigate the lipid composition in the peri-implantational period in the receptive uterus. In our study, we corroborated that the PC composition of maternal tissues in early pregnancy was different in IVF cases compared to a normal pregnancy (Figure 3, Supplementary Figures S1–S9).

Hamori et al. already described in the late 1980s that IVF can cause delayed implantation [29]. This explains the difference that can be detected in our images regarding the case of normal and IVF pregnancies (Figure 1).

Not only does the body extract the AA already mentioned from PLs, but many other free fatty acids are also released during the breakdown, which are known to have various effects both before and during pregnancy. In one of Shibahara et al.’s studies, the effect of palmitic acid (PA, C16:0) on maturing oocytes was investigated. It has been reported that a high PA concentration endangers the quality and developmental ability of the early embryo and can also lead to histone modification [30]. Aardema et al. reported a reduced developmental capacity after fertilization and a dose-dependent negative effect on the amount of fat stored in the lipid droplets of the oocyte in relation to PA and stearic acid (SA, C18:0). Furthermore, the opposite effect of monounsaturated oleic acid (OA, C18:1n-9) was reported, whereby OA was found to cause a slight increase in the lipid stored in lipid droplets and an improvement in post-fertilization development. These findings indicate that the ratio of saturated and unsaturated fatty acids has a significant impact on the developmental capability of the oocyte [31]. Oseikria et al. investigated the effect of a long-chain polyunsaturated fatty acid (LCPUFA), docosahexaenoic acid (DHA, C22:6n-3), on bovine oocytes and during IVF. When given at a low physiological concentration, DHA improved the oocyte cleavage rate, and the embryos reached the blastocyst stage to a greater extent. Administration of high concentrations of DHA had the exact opposite effect, as it disturbed the lipid metabolism of the surrounding cumulus cells [32]. Abolghasemi et al. in one of their publications wrote about the effect of dietary supplement linoleic acid (LA, C18:2n-6) on gene expression. An increase in the plasma progesterone level of cattle was found, the background of which was a reduced CB2 receptor production and N-acyl phosphatidylethanolamine phospholipase D (NAPE-PLD) enzyme synthesis in the endometrium [33].

In our study, PC34:1 and PC36:1, which are predominantly OA-containing, exhibited high intensities in different parts of the uterus during the peri-implantational period in both normal and IVF pregnancies. However, the accumulation of these lipids differed between the two groups. For instance, PC36:1 was observed in the mesometrial decidua in IVF pregnancy, but was almost absent in normal pregnancy. This striking difference suggests the possible role of OA-containing lipids in the early embryonal life, which may play a pathobiochemical role in the early embryonal development in IVF.

The polyunsaturated fatty acid-containing PCs demonstrated both spatial and temporal differences during the peri-implantational period in both groups. It is noteworthy that the greatest discrepancies between the normal and IVF groups were observed at embryonic day 8.5 in the intensity of these PC lipids (PC36:2, PC36:4, PC38:4, and PC 40:6). A maternal diet high in n-3 or n-6 fatty acids can effectively modify the fatty acid composition of uterine PLs and can even increase the implantation rate in mice [34]. A higher maternal DHA intake in women undergoing IVF has been shown to improve embryo morphology, suggesting a potential link between DHA and IVF success rates [35]. Furthermore, maternal LCPUFA-supply before implantation can increase the success of implantation, clinical pregnancy, and live birth. This is mainly due to the effects of n-3 LCPUFAs, such as eicosapentaenoic acid [36].

The present study confirmed that PCs in the uterus of normal pregnant mice undergo both temporal and spatial changes during the peri-implantation period in early pregnancy [4]. However, to the best of our knowledge, we are the first to investigate the changes in PCs with imaging mass spectrometry in an IVF model and to compare individual lipids with those of a normal pregnant uterus.

Given that PLs, primarily through the fatty acids they contain, play a pivotal role in numerous signalling processes, including uterine receptivity, optimal uterine–embryonic communication, implantation, and later placental development, it is crucial to elucidate the distinctions between the normal and IVF uterine lipid distribution patterns. The identification of the specific lipid classes and fatty acids involved during implantation may facilitate a deeper understanding of the biochemical mechanisms underlying implantation, thereby potentially improving the outcomes of IVF techniques.

## 4. Materials and Methods

### 4.1. Animals

In this study 54 age-matched female CD-1 mice were randomly assigned to two experimental groups (n = 27 in each). The first group of female mice (IVF group) were mated with infertile (vasectomised) and the second group (normal pregnant) with fertile males of the same strain to induce pseudopregnancy or pregnancy, respectively. Mice were euthanized between 0900 and 1000 h on the specified day of pregnancy (0 h on day 1 = vaginal plug). The 54 uteri samples (technical triplicates from each group) were collected from animals in the oestrus cycle, pseudopregnant and pregnant animals (natural and transferred embryos) at embryonic days 4.5, 6.5, 8.5, 10.5, 12.5, and 16.5, respectively.

Our mice were maintained on a 12 h light/dark cycle, on a standard rodent diet, and water was available as needed. Increased oocyte maturation was induced in 6–12-week-old CD-1 female mice using 5 IU intraperitoneally injected follicle-stimulating hormone (Meriofert, IBSA Pharma, Castagnola-Lugano, Switzerland). After 48 h, these mice were treated with 5 IU luteinizing hormone (Chloragon, Ferring, Budapest, Hungary). The next day, the females were humanely killed by cervical dislocation, and then the oviduct was dissected, and the cumulus–oocyte complex (COC) was collected. At the same time, sperm were collected from the cauda epididymis after humane cervical dislocation of an 8–12-week-old male CD-1 mouse. The sperm were incubated for 1 h in 200 µL of human tubal fluid (HTF) medium (Cosmo Bio, Tokyo, Japan) under mineral oil at 37 °C and 5% CO_2_ pressure until capacitation was complete. Female and male gametes were then encountered in 300 µL of HTF medium under mineral oil and incubated for 4 h at 37 °C with 5% CO_2_. The putative zygotes were placed in 50 µL of KSOM medium (Millipore, Watford, UK) under mineral oil and then incubated for an additional 3 days until they reached the blastocyst stage. Culture media were replaced every 24 h. At 3.5 days, embryos were transferred into pseudo-pregnant BL6/CBA F1 females. A total of 6–8 embryos were surgically transferred into both horns of the uterus.

### 4.2. Histological Tissue Sections

For routine histological investigations, 5 µm thick non-fixed 2% carboxymethyl cellulose embedded sections were made by Leica CM1860 UV cryostat (Biomarker Kft., Budapest, Hungary); the slides were stained with standard hematoxylin and eosin and documented using a Pannoramic Desk digital slide scanner (3D Histech, Budapest, Hungary) using Pannoramic Viewer 1.15 software for data evaluation.

### 4.3. MALDI Imaging Mass Spectrometry

Imaging mass spectrometry (IMS) offers the possibility to combine histological information with label-free mass spectrometric imaging technology.

Tissue samples were stored at −80 °C until processing. Freshly prepared 2% carboxymethyl cellulose embedding material was used for immobilization, and a Leica CM1860 cryostat (Leica Microsystems GmbH, Wetzlar, Germany) was applied at −19 °C for tissue sectioning. Tissues were cut at a thickness of 15 μm and thaw-mounted onto indium-tin-oxide-coated glass slides (Bruker Daltonics, Bremen, Germany). An Alpha-Cyano-4-hydroxycinnamic acid (CHCA, Bruker Daltonics, Bremen, Germany) matrix was applied in 70 deposition-drying cycles by using an automated piezoelectric spray device. The 7 mg/mL matrix solution was prepared fresh every day by dissolving CHCA in 6:4 acetonitrile—0.2% aqueous trifluoroacetic acid (TFA, Spectranal quality, Sigma-Aldrich, Budapest, Hungary). Mass spectra were acquired on an Autoflex Speed MALDI TOF/TOF mass spectrometer equipped with a 1 kHz Smartbeam-II solid-state laser (Bruker Daltonics, Bremen, Germany). The MALDI measurements were performed in positive reflectron mode in a detection range of *m*/*z* 400 to 3000. The lateral resolution for MALDI imaging was set to 50 μm. A total of 250 laser shots were summarized from each position. The acquisition and evaluation were carried out using FlexImaging 3.0 and FlexControl 3.4 software (Bruker Daltonics, Bremen, Germany). Lipid identification was carried out by MALDI TOF/TOF mass spectrometry using LIFT mode for PSD (post source decay) and CID (collision-induced decay) fragmentation.

### 4.4. Lipid Identification by HPLC-MS/MS

Microdissections from uteri of the non-pregnant and pregnant mice were used for identification of the lipids. Lipids from the mouse uterus were extracted with a modified Bligh and Dyer extraction method [37]. Two 100 µm tissue sections were loaded into a glass vial, and lipids were extracted by adding 1 mL extracting solution (chloroform/methanol/water 60/30/10 *v*/*v*%) containing Butylated hydroxytoluene (10 ng/mL) as an antioxidant. First, the samples were homogenized and vortexed for 2 min, then, we helped the extraction with ultrasound for 10 min. The samples were allowed to stand for 30 min, but we vortexed them every five minutes for 30 s. After the first extraction phase, the organic phase was removed, and we put it in a new glass vial. In the second extraction phase, 500 µL of extraction solution was added to the remains of the inorganic phase. We performed the extraction again, and the new organic phase was added to the first extraction. Next, we dried the extracted lipids under nitrogen gas, and we resolved them with 50 µL acetonitrile/isopropanol/water (65/30/5 *v*/*v*% in 0.1% formic acid).

Identification of lipid profiles was carried out by liquid chromatography coupled tandem mass spectrometry. Analyses were performed with a complex Ultimate 3000 (Dionex, Sunyvale, CA, USA) HPLC system equipped a Q-Exactive mass spectrometer with HESI source (ThermoFischer Scientific, Waltham, MA, USA). Separations were performed on a Kinetex C-18 (150 mm, 2.1 mm i.d., particle size 2.6 µm) column (Phenomenex, Torrance, CA, USA). The flow rate was 150 µL/min with a sample injection volume of 5 µL. Data acquisition and analysis were performed using Xcalibur 1.4 (ThermoFischer Scientific, Waltham, MA, USA). A binary gradient consisting of mobile phases A and B (A: methanol, 0.1% formic acid; B: water, 0.1% formic acid) was applied for the chromatographic separation. The compounds were separated with the following gradient profile: 40% A for 5 min followed by 10 min linear gradient to 90% and an isocratic period of 5 min. The column was equilibrated to the initial conditions with a 1 min linear gradient to 40% A and an isocratic period of 5 min. Both MS and MS/MS measurements were performed in the positive ion mode. The HESI source was operated with a spray voltage of 3500 V. Both sheath and auxiliary gases were nitrogen, delivered at 10 and 2 l min, respectively. The capillary temperature was set at 200 °C, while the HESI source heater was set at 40 °C. The RF level of the S-lenses was set at 70. MS/MS experiments were performed with an isolation window of *m*/*z* 2.0 where parent ions were *m*/*z* 306, 308, 482, and 484; normalized collision energy was set at 35 eV.

The results of the LC-MS/MS-based lipid maps database search are shown in Supplementary Figure S10.

## 5. Conclusions

In conclusion, this article presents a significant spatio-temporal change in phospholipid metabolism during the early stage of embryogenesis, which can greatly influence the success of IVF procedures or even influence the growing offspring. Further multimodal imaging investigations are needed for the discovery of detailed molecular mechanisms during implantation and early stage embryogenesis after assisted reproductive technology.

## Data Availability

Data are contained within the article.

## Supplementary Materials

The following supporting information can be downloaded at https://www.mdpi.com/article/10.3390/ijms25137423/s1.
