# Supplementary material for: Investigation of Phosphatidylcholine by MALDI Imaging Mass Spectrometry in Normal and IVF Early-Stage Embryos
Source: Int J Mol Sci. 2024 Jul 6;25(13):7423. doi: 10.3390/ijms25137423 (PMC11242196; doi:10.3390/ijms25137423)
Supplement: Supplementary file 1 [file ijms-25-07423-s001.zip › ijms-3051724-supplementary.pdf]

Supplementary Figure S1. Representative spatial distribution of PC 32:0 by MALDI imaging mass spectrometry in normal pregnancy (left) and after embryo transfer (right) in the embryo-containing uterus at embryonic day 6.5 (upper images), 8.5 (middle images) and 10.5 (bottom images). All scale bars: 2000 µm. (Color code: blue-low concentration, red-high concentration)

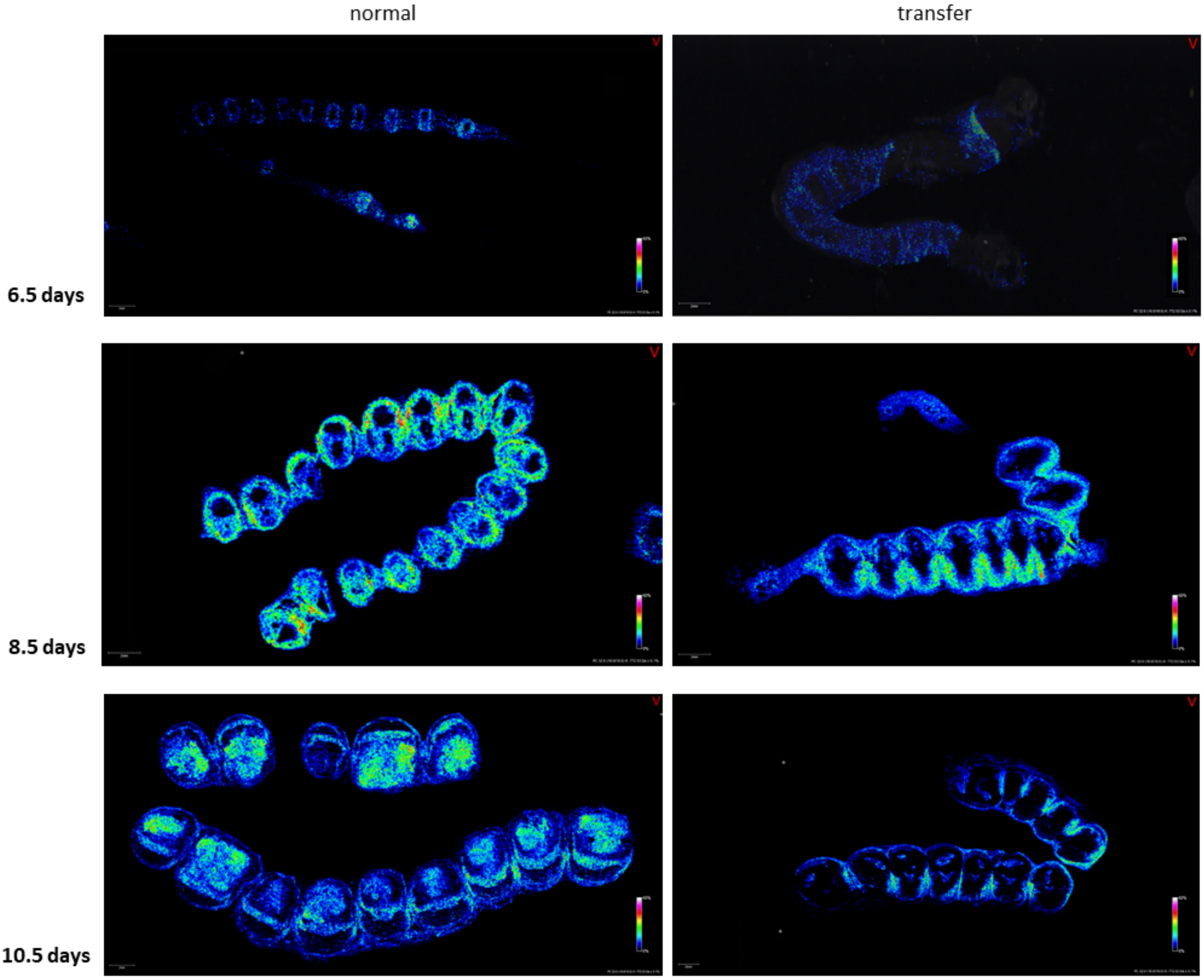

Supplementary Figure S2. Representative spatial distribution of PC 34:0 by MALDI imaging mass spectrometry in normal pregnancy (left) and after embryo transfer (right) in the embryo-containing uterus at embryonic day 6.5 (upper images), 8.5 (middle images) and 10.5 (bottom images). All scale bars: 2000 µm. (Color code: blue-low concentration, red-high concentration)

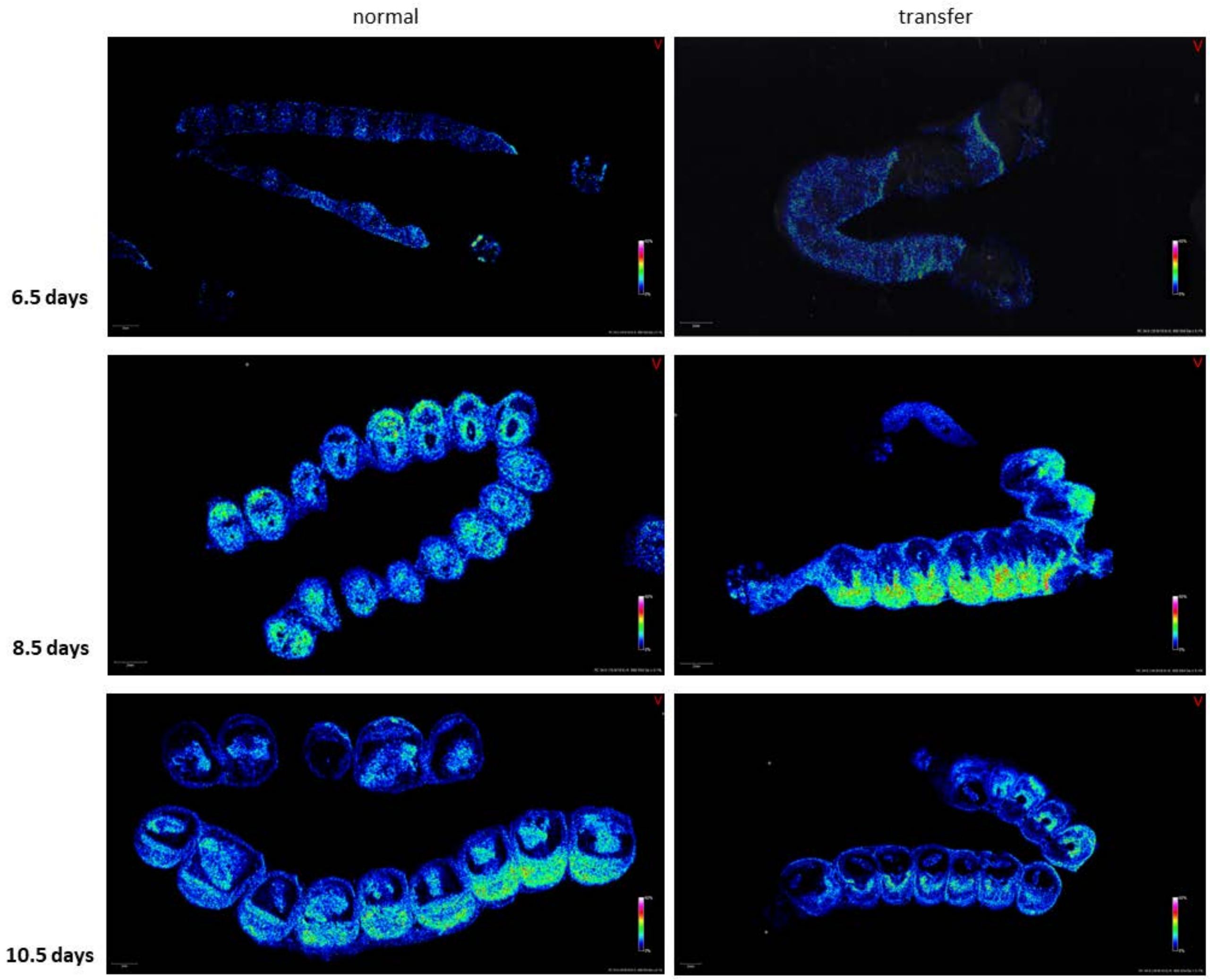

Supplementary Figure S3. Representative spatial distribution of PC 34:1 by MALDI imaging mass spectrometry in normal pregnancy (left) and after embryo transfer (right) in the embryo-containing uterus at embryonic day 6.5 (upper images), 8.5 (middle images) and 10.5 (bottom images). All scale bars: 2000 µm. (Color code: blue-low concentration, red-high concentration)

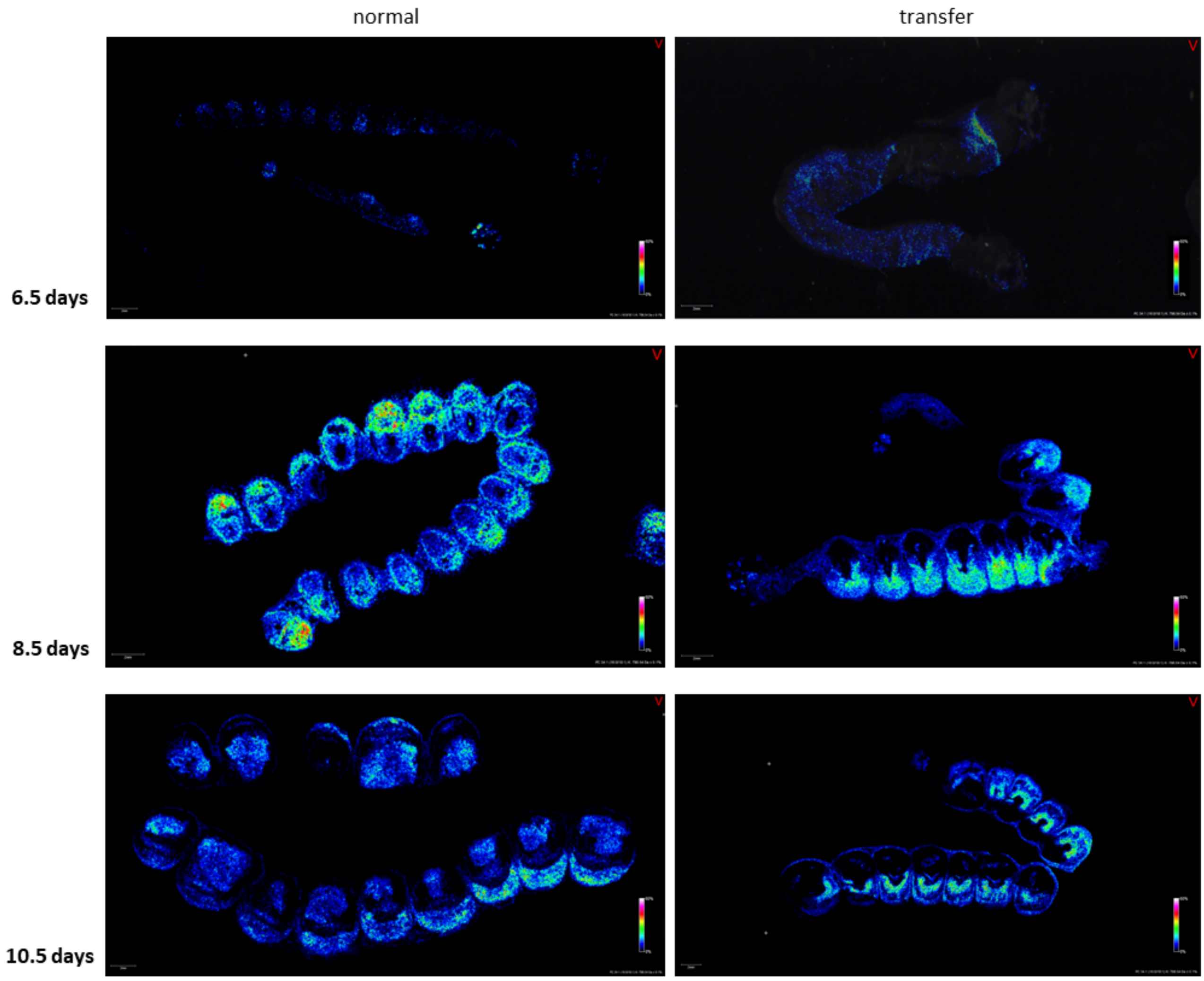

Supplementary Figure S4. Representative spatial distribution of PC 34:2 by MALDI imaging mass spectrometry in normal pregnancy (left) and after embryo transfer (right) in the embryo-containing uterus at embryonic day 6.5 (upper images), 8.5 (middle images) and 10.5 (bottom images). All scale bars: 2000 µm. (Color code: blue-low concentration, red-high concentration)

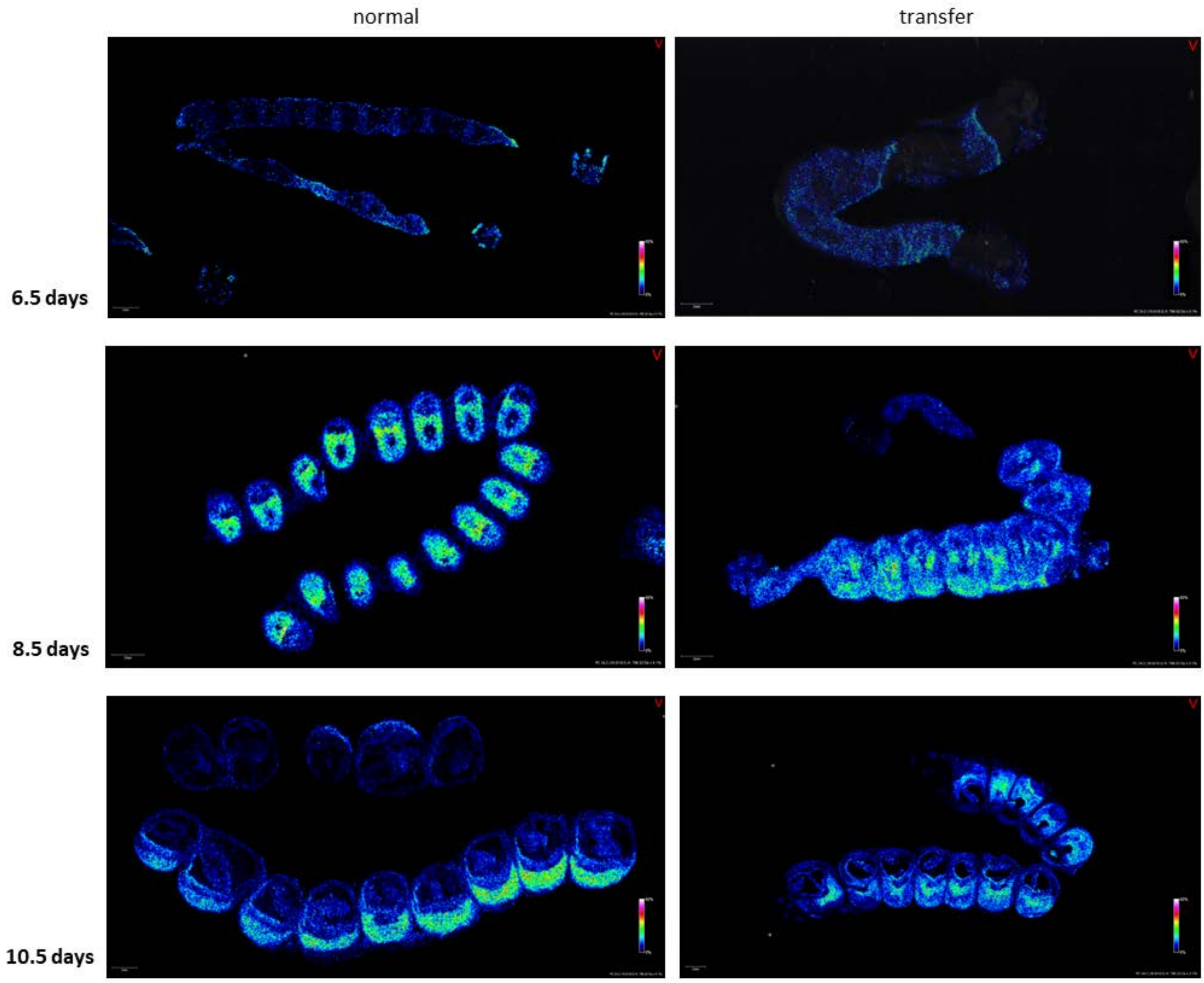

Supplementary Figure S5. Representative spatial distribution of PC 36:1 by MALDI imaging mass spectrometry in normal pregnancy (left) and after embryo transfer (right) in the embryo-containing uterus at embryonic day 6.5 (upper images), 8.5 (middle images) and 10.5 (bottom images). All scale bars: 2000 μm. (Color code: blue-low concentration, red-high concentration)

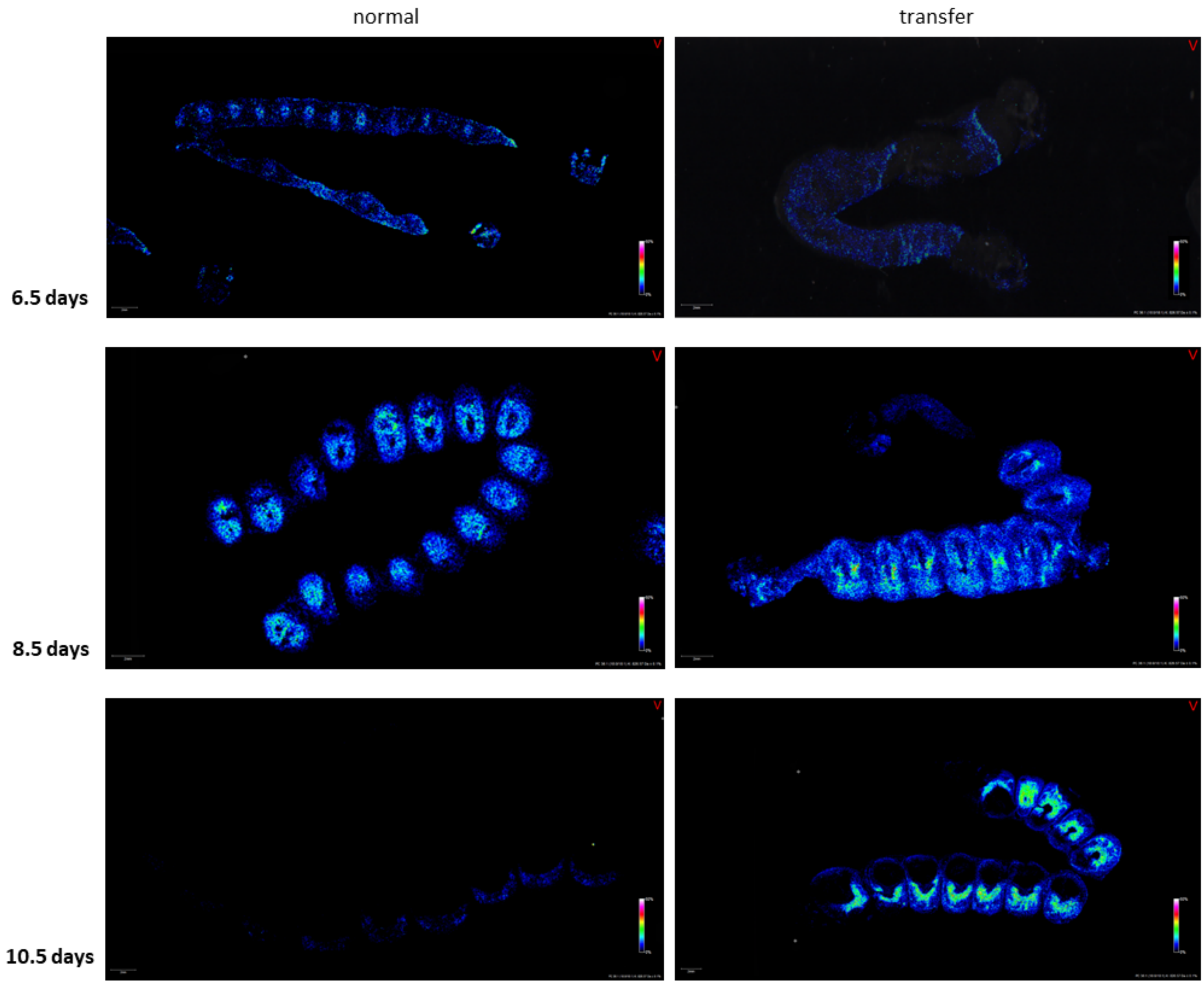

Supplementary Figure S6. Representative spatial distribution of PC 36:2 by MALDI imaging mass spectrometry in normal pregnancy (left) and after embryo transfer (right) in the embryo-containing uterus at embryonic day 6.5 (upper images), 8.5 (middle images) and 10.5 (bottom images). All scale bars: 2000 µm. (Color code: blue-low concentration, red-high concentration)

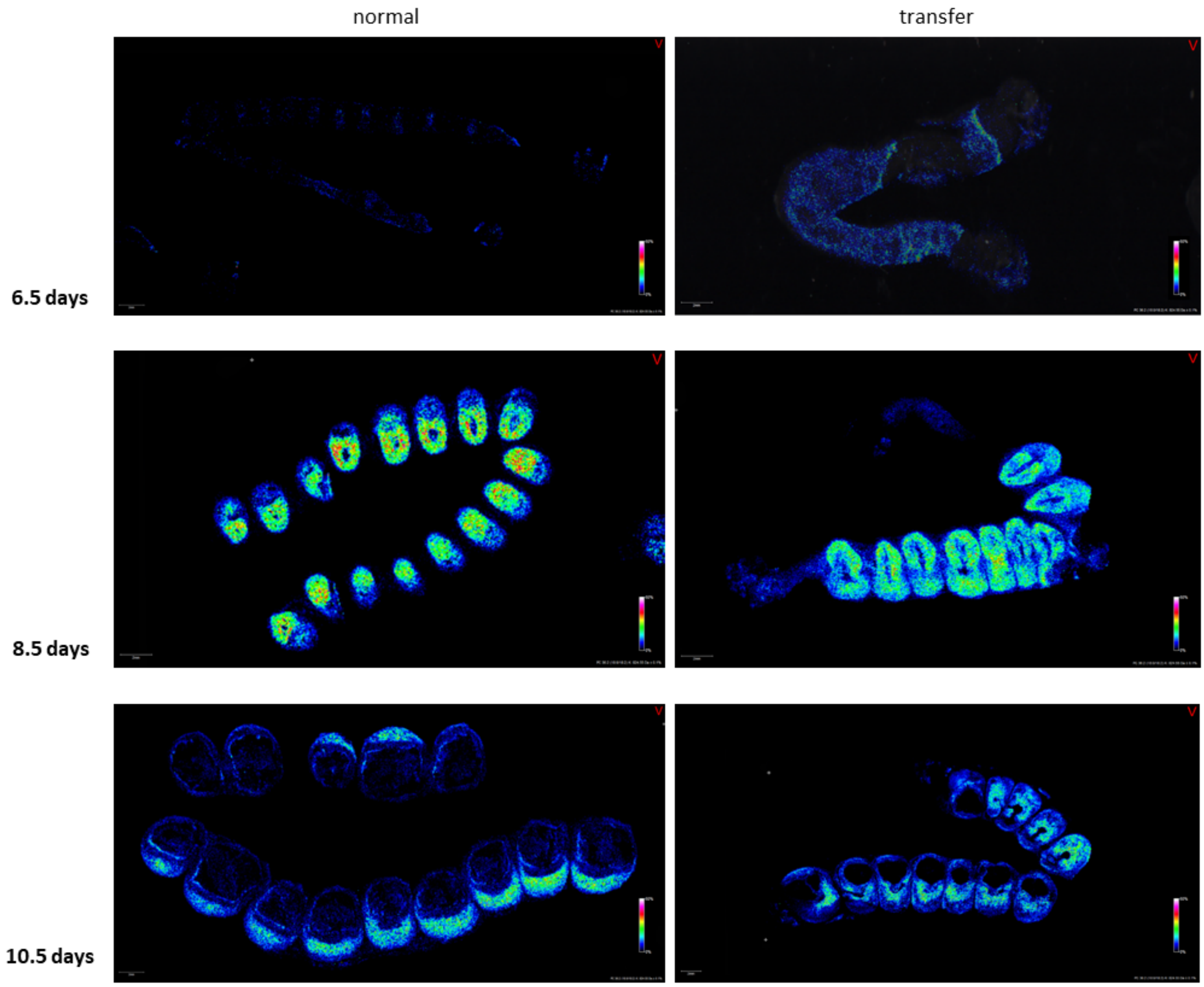

Supplementary Figure S7. Representative spatial distribution of PC 36:4 by MALDI imaging mass spectrometry in normal pregnancy (left) and after embryo transfer (right) in the embryo-containing uterus at embryonic day 6.5 (upper images), 8.5 (middle images) and 10.5 (bottom images). All scale bars: 2000 µm. (Color code: blue-low concentration, red-high concentration)

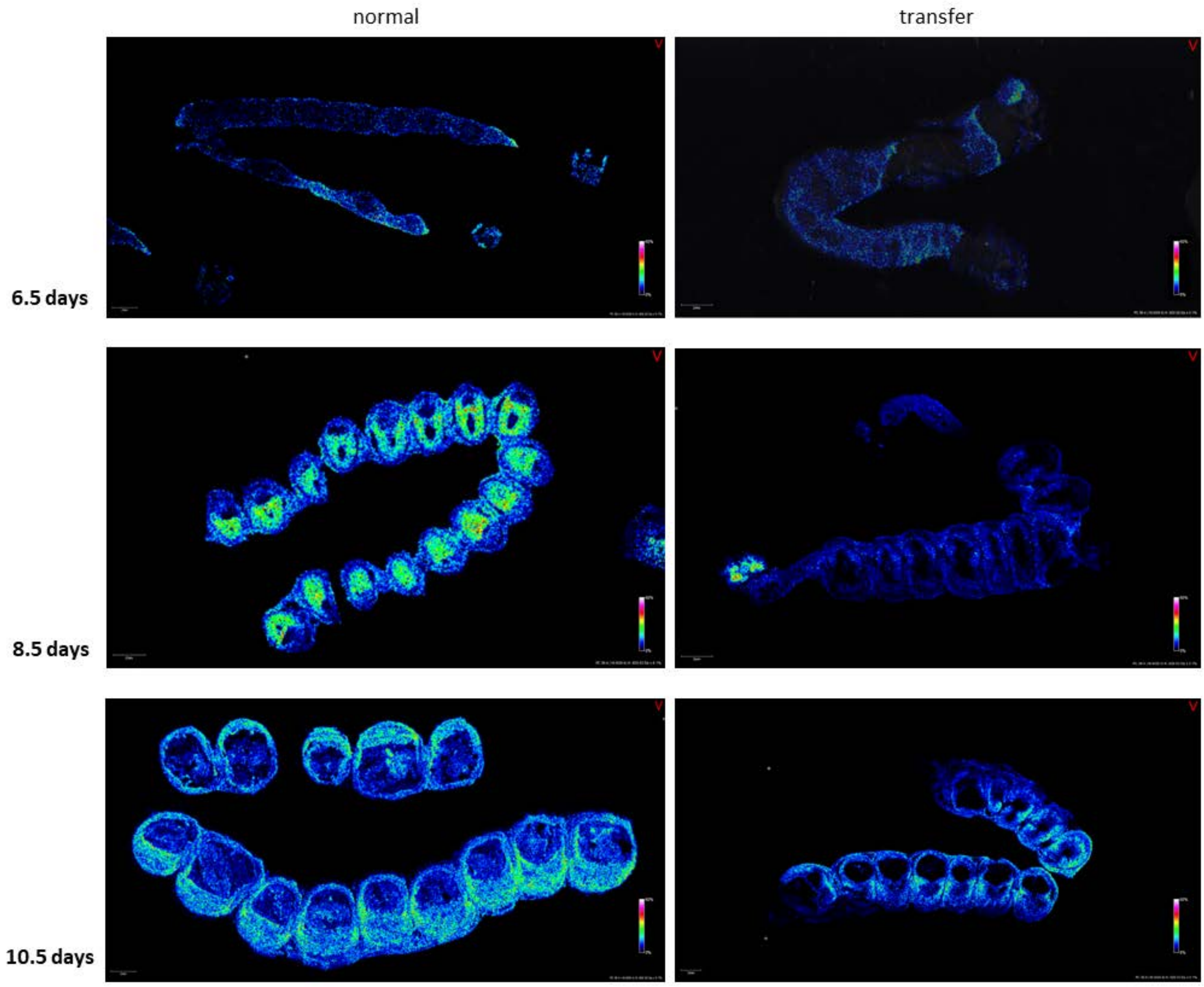

Supplementary Figure S8. Representative spatial distribution of PC 38:4 by MALDI imaging mass spectrometry in normal pregnancy (left) and after embryo transfer (right) in the embryo-containing uterus at embryonic day 6.5 (upper images), 8.5 (middle images) and 10.5 (bottom images). All scale bars: 2000 µm. (Color code: blue-low concentration, red-high concentration)

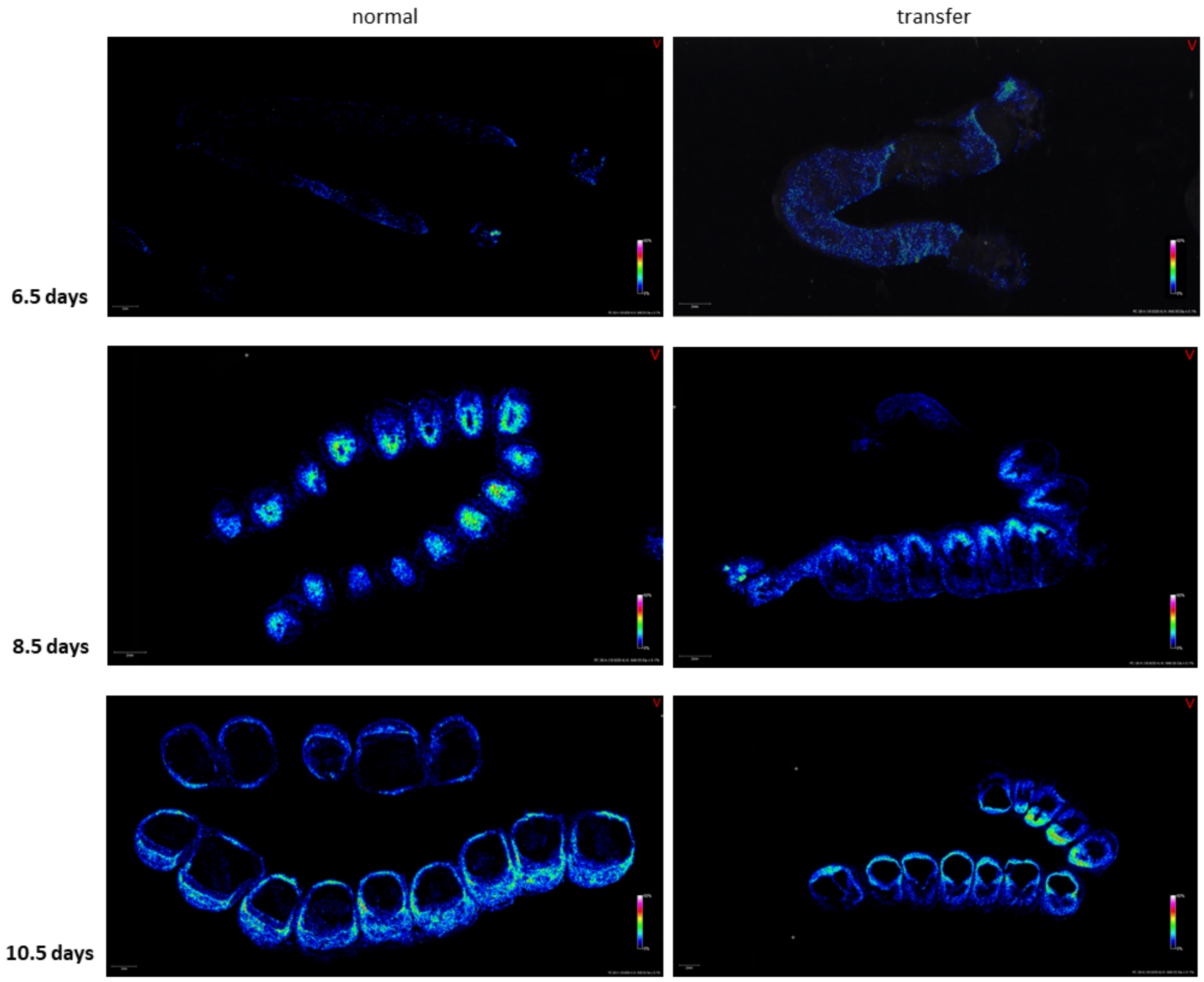

Supplementary Figure S9. Representative spatial distribution of PC 40:6 by MALDI imaging mass spectrometry in normal pregnancy (left) and after embryo transfer (right) in the embryo-containing uterus at embryonic day 6.5 (upper images), 8.5 (middle images) and 10.5 (bottom images). All scale bars: 2000 µm. (Color code: blue-low concentration, red-high concentration)

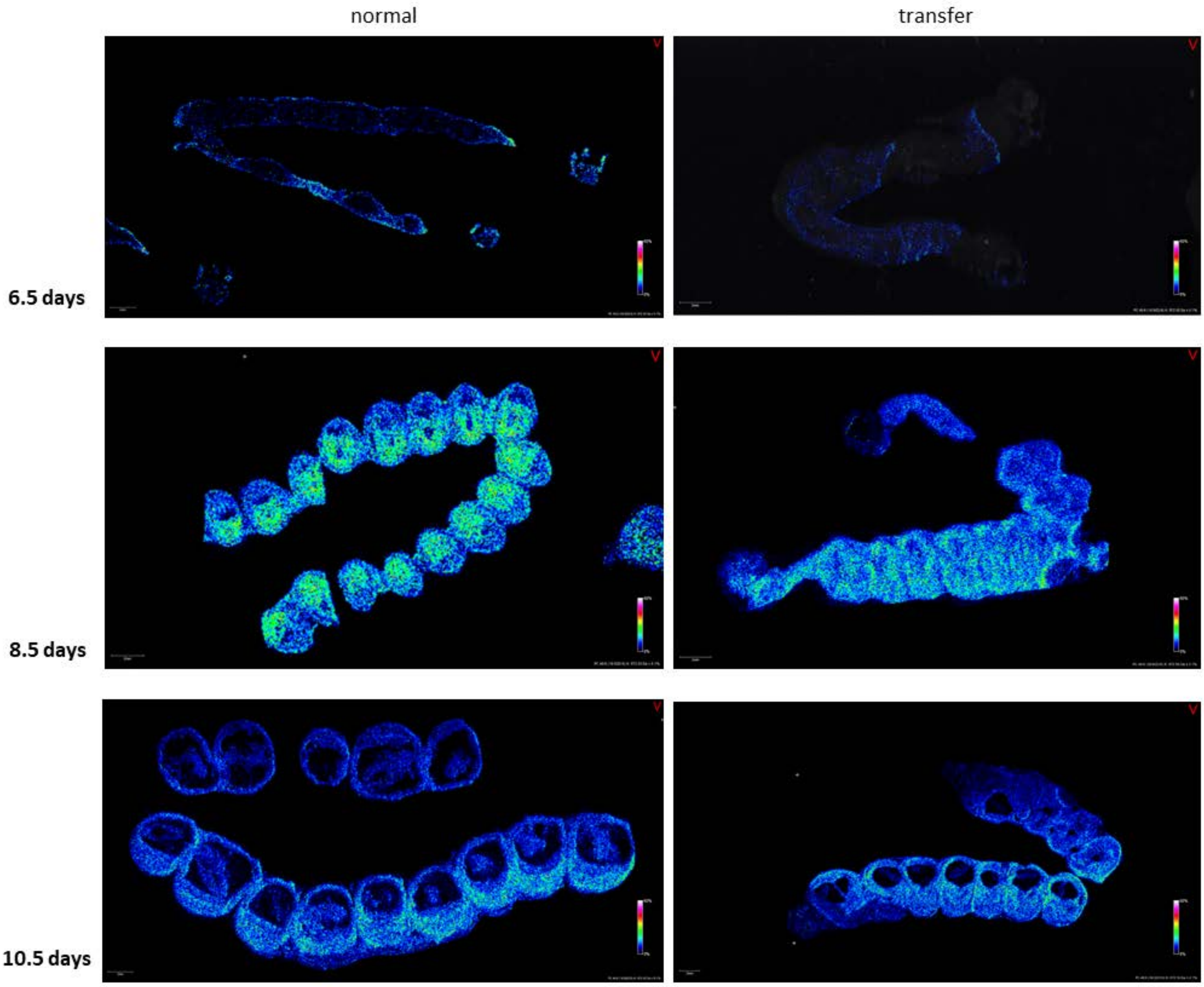

Supplementary Figure S10

lipid01

t ret	Modifications	Formula		Exact mass	Observed mass
**2.48_507.3700n**	M+H, M+Na	C26H54NO6P	PC(P-18:0/0:0)	507.3700471	508.3773235
**2.17_572.3721m/z**	M+H	C30H54NO7P	PC(22:4(7Z,10Z,13Z,16Z)/0:0)		572.3721438
**6.00_608.4669m/z**	M+H	C32H66NO7P	PC(24:0/0:0)		608.466925
**1.93_567.3330n**	M+H, M+Na	C30H50NO7P	PC(22:6(4Z,7Z,10Z,13Z,16Z,19Z)/0:0)	567.3329573	568.3402337
**4.38_579.4290n**	M+Na, M+K	C30H62NO7P	PC(22:0/0:0)	579.4290071	602.4182278
**1.99_543.3336n**	M+H, M+Na	C28H50NO7P	PC(20:4(5Z,8Z,11Z,14Z)/0:0)	543.3335545	544.340831
**3.29_551.3974n**	M+H, M+Na	C28H58NO7P	PC(20:0/0:0)	551.3974234	574.3874067
**2.54_523.3652n**	M+H, M+K, M+Na	C26H54NO7P	PC(18:0/0:0)	523.365207	524.3724835
**2.11_495.3337n**	M+H-H2O, M+K, M+H, M+Na	C24H50NO7P	PC(16:0/0:0)	495.3336752	496.3412131
**16.41_824.6170m/z**	M+H	C47H86NO8P	PC(17:0/22:4(7Z,10Z,13Z,16Z))		824.6169597
**14.24_793.5627n**	M+H, M+Na	C45H80NO8P	PC(15:1(9Z)/22:4(7Z,10Z,13Z,16Z))	793.5626826	794.569959
**14.96_793.5634n**	M+H, M+Na	C45H80NO8P	PC(15:1(9Z)/22:4(7Z,10Z,13Z,16Z))	793.5633635	794.5706399
**13.50_793.5642n**	M+H, M+Na	C45H80NO8P	PC(15:1(9Z)/22:4(7Z,10Z,13Z,16Z))	793.5642299	794.5715064
**17.12_798.6030m/z**	M+H	C45H84NO8P	PC(15:1(9Z)/22:2(13Z,16Z))		798.6029794
**16.12_798.6020m/z**	M+H	C45H84NO8P	PC(15:1(9Z)/22:2(13Z,16Z))		798.6020284
**12.46_791.5479n**	M+H, M+Na, M+K	C45H78NO8P	PC(15:0/22:6(4Z,7Z,10Z,13Z,16Z,19Z))	791.5479355	792.555212
**13.37_791.5475n**	M+H, M+Na	C45H78NO8P	PC(15:0/22:6(4Z,7Z,10Z,13Z,16Z,19Z))	791.5475094	792.5547859
**14.05_770.5715m/z**	M+H	C43H80NO8P	PC(15:0/20:3(8Z,11Z,14Z))		770.5715084
**14.66_770.5718m/z**	M+H	C43H80NO8P	PC(15:0/20:3(8Z,11Z,14Z))		770.5717542
**9.51_763.5170n**	M+H, M+Na	C43H74NO8P	PC(13:0/22:6(4Z,7Z,10Z,13Z,16Z,19Z))	763.5170433	764.5243197
**10.59_763.5172n**	M+H, M+Na	C43H74NO8P	PC(13:0/22:6(4Z,7Z,10Z,13Z,16Z,19Z))	763.5171834	764.5244599
**15.28_772.5858m/z**	M+H	C43H82NO8P	PC(13:0/22:2(13Z,16Z))		772.5858159
**10.90_739.5168n**	M+H, M+Na, M+K	C41H74NO8P	PC(13:0/20:4(5Z,8Z,11Z,14Z))	739.5168479	740.5241244
**9.93_739.5173n**	M+H, M+Na, M+K	C41H74NO8P	PC(13:0/20:4(5Z,8Z,11Z,14Z))	739.5172513	740.5245277
**10.53_742.5368m/z**	M+H	C41H76NO8P	PC(13:0/20:3(8Z,11Z,14Z))		742.5368447
**11.44_741.5342n**	M+H, M+Na	C41H76NO8P	PC(13:0/20:3(8Z,11Z,14Z))	741.5341927	742.5414692
**9.93_716.5257m/z**	M+H	C39H74NO8P	PC(13:0/18:2(9Z,12Z))		716.5257247
**10.90_715.5186n**	M+H, M+Na	C39H74NO8P	PC(13:0/18:2(9Z,12Z))	715.5186303	716.5259067
**9.93_790.5411m/z**	M+H	C45H76NO8P	PE(18:1(9Z)/22:6(4Z,7Z,10Z,13Z,16Z,19Z))		790.541124
**13.37_767.5468n**	M+H, M+K, M+Na	C43H78NO8P	PC(18:2(9Z,12E)/17:2(9Z,11E))	767.5468111	768.5540876
**13.69_767.5469n**	M+H, M+Na, M+K	C43H78NO8P	PC(18:2(9Z,12E)/17:2(9Z,11E))	767.5468578	768.5541343
**12.58_834.5996m/z**	M+H	C48H84NO8P	PC(18:0/22:6(4Z,7Z,10Z,13Z,16Z,19Z))		834.5996172
**16.53_822.6004m/z**	M+H	C47H84NO8P	PC(16:0/23:5(8E,11E,14E,17E,20E))		822.6004435
**15.40_822.6023m/z**	M+H	C47H84NO8P	PC(16:0/23:5(8E,11E,14E,17E,20E))		822.6022887
**9.81_805.5623n**	M+H, M+Na	C46H80NO8P	PC(16:0/22:6(4E,7E,10E,13E,16E,19E))	805.5623302	806.5696067
**13.37_744.5563m/z**	M+H	C41H78NO8P	PC(15:0/18:2(9Z,12Z))		744.5563318
**13.69_744.5565m/z**	M+H	C41H78NO8P	PC(15:0/18:2(9Z,12Z))		744.5564927
**15.40_746.5714m/z**	M+H	C41H80NO8P	PC(15:0/18:1(11Z))		746.5713911
**14.91_746.5705m/z**	M+H	C41H80NO8P	PC(15:0/18:1(11Z))		746.5704894
**8.88_754.5367m/z**	M+H	C42H76NO8P	PC(14:0/20:4(5Z,8Z,11Z,14Z))		754.5366782
**11.80_754.5409m/z**	M+H	C42H76NO8P	PC(14:0/20:4(5Z,8Z,11Z,14Z))		754.5409031
**9.39_754.5376m/z**	M+H	C42H76NO8P	PC(14:0/20:4(5Z,8Z,11Z,14Z))		754.5376132
**14.96_795.5784n**	M+H, M+Na, M+K	C45H82NO8P	PC(17:0/20:4(5Z,8Z,11Z,14Z))	795.5783918	796.5856682
**15.58_795.5795n**	M+H, M+K, M+Na	C45H82NO8P	PC(17:0/20:4(5Z,8Z,11Z,14Z))	795.5794813	796.5867577
**12.71_717.5336n**	M+H, M+Na	C39H76NO8P	PC(16:0/15:1(14))	717.5336312	718.5409076
**12.28_717.5326n**	M+H, M+Na	C39H76NO8P	PC(16:0/15:1(14))	717.5326185	718.539895
